# Using queueing models as a decision support tool in allocating point-of-care HIV viral load testing machines in Kisumu County, Kenya

**DOI:** 10.1093/heapol/czad111

**Published:** 2023-11-09

**Authors:** Yinsheng Wang, Anjuli D Wagner, Shan Liu, Leonard Kingwara, Patrick Oyaro, Everlyne Brown, Enerikah Karauki, Nashon Yongo, Nancy Bowen, John Kiiru, Shukri Hassan, Rena Patel

**Affiliations:** Department of Global Health, University of Washington, Seattle, Washington 98195, USA; Department of Industrial and Systems Engineering, University of Washington, Seattle, Washington 98195, USA; Department of Industrial and Systems Engineering, University of Washington, Seattle, Washington 98195, USA; National Public Health Laboratory, Nairobi 00100, Kenya; LVCT Health, Mombasa 80100, Kenya; UW Kenya, Nairobi 00100, Kenya; UW Kenya, Nairobi 00100, Kenya; UW Kenya, Nairobi 00100, Kenya; National Public Health Laboratory, Nairobi 00100, Kenya; National Public Health Laboratory, Nairobi 00100, Kenya; Department of Global Health, University of Washington, Seattle, Washington 98195, USA; Department of Global Health, University of Washington, Seattle, Washington 98195, USA; Division of Allergy and Infectious Diseases, Department of Medicine, University of Washington, Seattle 98195, USA; Division of Infectious Diseases, Department of Medicine, University of Alabama at Birmingham, Birmingham, Alabama 35294, USA

**Keywords:** HIV viral load, policymaker, decision support, modelling, point-of-care

## Abstract

Point-of-care (POC) technologies—including HIV viral load (VL) monitoring—are expanding globally, including in resource-limited settings. Modelling could allow decision-makers to consider the optimal strategy(ies) to maximize coverage and access, minimize turnaround time (TAT) and minimize cost with limited machines. Informed by formative qualitative focus group discussions with stakeholders focused on model inputs, outputs and format, we created an optimization model incorporating queueing theory and solved it using integer programming methods to reflect HIV VL monitoring in Kisumu County, Kenya. We modelled three scenarios for sample processing: (1) centralized laboratories only, (2) centralized labs with 7 existing POC ‘hub’ facilities and (3) centralized labs with 7 existing and 1–7 new ‘hub’ facilities. We calculated total TAT using the existing referral network for scenario 1 and solved for the optimal referral network by minimizing TAT for scenarios 2 and 3. We conducted one-way sensitivity analyses, including distributional fairness in each sub-county. Through two focus groups, stakeholders endorsed the provisionally selected model inputs, outputs and format with modifications incorporated during model-building. In all three scenarios, the largest component of TAT was time spent at a facility awaiting sample batching and transport (scenarios 1–3: 78.7%, 89.9%, 91.8%) and waiting time at the testing site (18.7%, 8.7%, 7.5%); transportation time contributed minimally to overall time (2.6%, 1.3%, 0.7%). In scenario 1, the average TAT was 39.8 h (SD: 2.9), with 1077 h that samples spent cumulatively in the VL processing system. In scenario 2, the average TAT decreased to 33.8 h (SD: 4.8), totalling 430 h. In scenario 3, the average TAT decreased nearly monotonically with each new machine to 31.1 h (SD: 8.4) and 346 total hours. Frequency of sample batching and processing rate most impacted TAT, and inclusion of distributional fairness minimally impacted TAT. In conclusion, a stakeholder-informed resource allocation model identified optimal POC VL hub allocations and referral networks. Using existing—and adding new—POC machines could markedly decrease TAT, as could operational changes.

Key messages

- A stakeholder-informed resource allocation queueing model identified optimal point-of-care viral load (POC VL) machine hub allocations and referral networks in one Kenyan county.
- While adding POC machines would decrease turnaround time (TAT), larger reductions in TAT could be achieved by more frequent batching of samples and increasing the capacity of existing central labs and POC VL hubs.

## Introduction

HIV viral load (VL) monitoring is crucial for optimizing health outcomes for persons living with HIV. Existing standard-of-care (SOC), centralized laboratory-based systems for VL monitoring have improved globally, but many still face substantial challenges. Delays occurring at multiple levels, including at sample request, collection and processing, results release, return and review and clinical action culminate in the sub-optimal identification of patients lacking viral suppression. As countries globally work towards achieving the Joint United Nations Programme on HIV/AIDS 95–95-95 goals for HIV testing, treatment and virological suppression, point-of-care (POC), or even near POC, technologies have played a major role, particularly in high HIV prevalence, resource-limited settings (Pai *et al.*, 2015). POC tests are simple to use, reduce test turnaround time (TAT), increase receipt of results, accelerate clinical decision-making and improve clinical management (Menendez-Botet, 2003). They overcome barriers related to sample transportation time and distance and cost, results return, patient receipt of results and subsequent clinical decision-making (Pai *et al.*, 2012; Engel *et al.*, 2015; Nichols *et al.*, 2019), improving overall patient health (Drain *et al.*, 2020). POC diagnostic testing has been shown to be faster than SOC for early infant diagnosis (Meggi *et al.*, 2018), increasing the odds of linkage to care and reducing time to testing and CD4 results receipt (Wynberg *et al.*, 2014). Studies testing POC VL testing have mixed results in terms of virological suppression (Drain *et al.*, 2020; Patel *et al.*, 2022), but have consistently demonstrated reduced TAT and feasibility.

Strategic placement of POC technologies within structured sharing networks may expand the impact of limited numbers of machines in the face of resource shortages. Many POC technologies require substantial upfront resource investment, thus, POC machines may not be feasibly available at all facilities that could benefit from this technology. Placement of POC technologies within networks—including ‘hub-and-spoke’ structures, in which one facility (‘hub’) has a stationary POC machine and receives samples from neighbouring facilities (‘spokes’)—can explicitly increase the reach and potential impact of such a technology. Such sharing networks allow for broader reach of innovative technology, but are complex by their nature. Modelling allows decision-makers to consider the optimal strategy, or combination of strategies, to maximize coverage, minimize TAT and minimize cost. Ministries of health have experience creating and utilizing models to inform decisions related to resource allocation and may be willing to expand this practice.

While the application of system engineering tools is well-established in resource-rich contexts, there is paucity of data for their use in resource-limited contexts, where results may have large potential to direct the use of limited resources (Wagner *et al*., 2019). Specifically, queueing models, which are a mathematical study of waiting lines to predict waiting times and queue length, have been used extensively in resource-rich settings, particularly to plan emergency department and hospital admission patterns, resource allocation and staffing (Hagen *et al.*, 2013; Thomas *et al.*, 2013; van Eeden *et al.*, 2016) for HIV care in the USA (Liu *et al.*, 2016; Gonsalves *et al.*, 2017). While studies of queues and waiting time in sub-Saharan Africa are common (Alamo *et al.*, 2013; Ameh *et al.*, 2013; Monroe-Wise *et al.*, 2017; Hoffmann *et al.*, 2018; Pintye *et al.*, 2018), use of queueing models to inform clinical improvement at systems level is rare and novel. Related efforts to understand the optimal distribution and storage of vaccines and HIV treatment using operational research have been successful in improving care in resource-poor environments (Alistar *et al.*, 2014; Haidari *et al.*, 2015; Hirsh Bar Gai *et al.*, 2018).

In this study, we aimed to develop and use a queueing model in companion with an optimization model to address the complex system challenges of POC VL monitoring machine allocation within Kisumu County, Kenya, one of Kenya’s regions with the largest burden of HIV. We first conducted formative research with focus group discussions (FGDs) among policymakers and then developed a queueing model to solve for the optimal machine allocation under three scenarios.

## Methods

### Formative data collection

We undertook formative qualitative research utilizing FGDs to understand Kenyan policymakers’ preferences for model function and decision-making factors. The goal was to inform the modelling inputs, outputs and format. The topics in the question guide included: decision-making factors related to placement of POC technology generally and specifically for VL monitoring, factors used in prioritizing VL POC machines, and model inputs and outputs. A set of a priori suggested model inputs were offered for reflection, based on a Kenyan early infant diagnosis POC resource allocation model *(Lusike, Wafula, Kingwara, Kenya POC Placement Model, 2018),* co-authors’ understanding of VL testing systems in Kenya and engineering co-authors’ expertise on essential inputs. We used a rapid qualitative coding approach following the framework methodology—a highly deductive matrix-based coding approach (Srivastava and Thomson, 2009; Gale *et al.*, 2013). The categories in the coding matrix were drawn from the topics of the question guide and included: factors that inform decision-making generally, factors to inform placement of POC VL machines, including what factors inform prioritization of placement, and model inputs and outputs. The results from the matrices were summarized into a narrative memo.

We recruited policymakers from county Ministry of Health teams, implementing partners and laboratory leaders. FGDs were conducted on Zoom in English, by a single, trained facilitator, and audio-recorded following oral informed consent and demographic characteristic survey completion.

### Model overview

We developed a queueing–location–allocation model based on queueing theory and integer programming (Hilier and Lieberman, 2015). The objective of the model is to: (1) minimize the TAT (operationalized as the total time at a facility prior to being sent to a processing centre [batching delay] and transportation time) of HIV VL testing for the whole system, and (2) to ensure each testing location is below 90% utilization while meeting all testing demand in Kisumu County. Decision variables include the selection of testing hubs from a list of existing HIV treatment facilities and three central labs and the referral network (i.e. which clinic sends samples to which hub or central lab in a hub-and-spoke model).

In section 2.1, we briefly introduce the problem including the facility setting, network scenarios and the basics of the queueing model. In section 2.2, we describe the data sources utilized to estimate testing demand, transportation distance and time between facilities and hubs and the parameterization of each queue at the testing site. In section 2.3, we describe how the optimization of the referral network is formulated and how the optimization problem is solved. In section 2.4, we describe the layout of a user-friendly decision-support Excel tool for policymakers.

### Problem overview

#### Facility setting

We modelled the process of HIV VL testing in Kisumu County to support policymakers to make decisions on the optimal placement of POC machines at facilities and identify the optimal transportation network of referring facilities. In Kisumu County’s healthcare system, there are 146 healthcare facilities that collect HIV VL testing samples. After collecting samples from patients, each facility sends their samples to one of the three central labs for testing (current SOC with large capacity testing equipment); 7 other facilities contain a POC machine (hubs) for tuberculosis diagnostics (described below).

While there are several existing POC machines that can process HIV VL samples, the most commonly available in Kenya is the GeneXpert^®^ platform, initially invested for decentralized tuberculosis testing in the country. The POC machine in this modelling exercise was based on a single GeneXpert^®^ machine with the four-cartridge module and a 90 min cycle for testing. Among the existing 7 facilities with these POC machines (hubs), most have only one or two such machines. We provide a flowchart of the HIV VL POC testing system with details in Figure A1 in Supplementary Appendix 2.

#### Scenario considerations

We considered three different network scenarios. Scenario 1 models a referral network with the 3 central labs only. Scenario 2 models the current situation involving 7 existing hubs and 3 central labs with an optimized referral network. Scenario 3 aims to provide insights to policymakers when they are planning to expand the current network by adding a maximum of seven additional POC hubs to the existing hubs and central labs. Additional details are provided in Table A1 in Supplementary Appendix 1 and Figure A2 in Supplementary Appendix 2.

#### Queueing models

We used queueing models to analyse the processing time in each testing facility (hub or centralized lab). Queues (waiting lines) are an important part of our everyday life. Queueing theory uses queueing models to represent various types of systems that involve ‘waiting in lines’, and study the performance of systems under given assumptions. M/M/s is one of the most widely studied queueing models (Hilier and Lieberman, 2015). Here we used a M/M/s queue to model the arrival and processing of VL testing samples at each selected hub in Kisumu County and at each central lab in Kenya. Details of the M/M/s queueing model can be found in Supplementary Appendix 3. Of note, we built two separate queues to reflect the processes at centralized labs, (1) entering samples into the computer system by the data or laboratory staff, and (2) sample preparation and testing process. The sum of these two queueing times is the expected time in the system.

### Data acquisition

In collaboration with Kenyan policymakers, researchers and laboratory specialists, our team collected model parameter information. All model parameter values and their data sources are listed in Table 1.

**Table 1.** Model parameters, assumptions and data sources

Parameter	Base case value	Note
VL test demand (per working day)		
Demand adjustment factor (average number of VL tests expected per person per year)	1.08	To estimate the number of VL samples from each clinic, we utilized DHIS II data for Kisumu County’s HIV client volume data from 2019. This was due to disruptions during the COVID-19 pandemic in 2020 and nationwide disruptions in VL testing afterwards.
Testing demand in 146 clinics	Ranges from 0 to 37 (per working day) for different clinics	We first calculated a weighted average of annual testing volume at each facility to reflect the expected average annual testing demand based on national guidelines in effect in 2022. Guidelines recommend testing children 2× per year. Adults newly starting treatment receive tests every 6 months for the first year, followed by annually thereafter, for an average of 1.08 tests per year. The equation for estimating annual testing volume is as follows: *Annual Testing Demand = 2 *×* Children volume per year + 1.08 *×* Adult volume per year* We then calculated the average daily demand at each facility assuming 20 workdays per month. Data Source: http://kmhfl.health.go.ke/ and https://dhis2.org/
Transportation		
Pairwise distance between all facilities (km)	0 to 141	We used Google Map API to collect the distance and time data given the name of facilities in Kisumu, Kenya. This involves two steps: matching facility names with its geo-location (longitude and latitude) and getting the pairwise distance between all facilities. (https://developers.google.com/maps)
Speed (km/h)	5 (walk), 20 (bike), 40 (motorbike)^a^ and 50 (car)	To calculate the transportation time, we provided different types of transportation modes, allowed the user to decide which one to use and estimated the average speed for each transportation mode.
Road condition adjustment coefficient Weather condition adjustment coefficient	0.8 (good), 1 (average)^a^ and 1.2 (bad) 0.8 (good), 1 (average)^a^, 1.2 (bad)	We considered different weather and road conditions and allowed users to change these conditions based on their needs. The weather and road conditions are ‘good’, ‘average’ or ‘bad’, and the time needed for transportation could be less given better weather and road conditions.
Batching delay (min): [frequency with which samples are transported to testing facility (hub or central lab)]		
Daily	210	Here and in later estimation, we assumed that each working day has 7 h. If the samples are sent daily, the expected delay time is half of the working day, which is 3.5 (h), i.e. 210 min.
Twice a week^a^	1860	If the samples are sent twice a week, the expected delay is 24 + 7 = 31 h, i.e. 1860 min.
Once a week	3090	If the samples are sent only once a week, the expected delay is ((4*24)+ 7)/2 =51.5 h, i.e. 3090 min.
Central lab queueing parameters		
Entering process		
Mean service rate (test per day) [Average number of VL samples processed at one central lab given current staffing and process steps]	710	We adjusted our central lab daily testing capacity to 710 as an adjusted assumption; in our original set of assumptions, the demand for testing exceeded the capacity in Scenario 1 and the queue exploded. This aligns with the common experience of patients who have to wait for an extremely long time before they receive their results. Our model also shows that when the mean service rate (number of tests completed in a workday) is increased to 692 samples per day with two servers in KEMRI CDC HIV/R Lab, then the queueing system could handle the current demand.
Number of servers (Number of workers processing the entering of samples)	2	We assume that there are two workers in each central lab working on entering the samples into the system.
Machine process		
Mean service rate (test per day)	1500	We assume each central lab can handle up to 1500 samples per day, and samples are handled in batches of 90 to be parallel processed in the machine. Estimates based on personal communication with central laboratory managers.
Number of machines at each central lab	1 (with 4 cartridges)	We assume the machine can process 90 samples in a batch, and each batch is processed in series. Estimates based on personal communication with central laboratory managers.
Percentage of samples from Kisumu		
KEMRI CDC	24% = 80 335/332 060	Since the central labs have large capacity for testing, they also accept samples from other counties around the country. We estimated the percentage of samples coming from Kisumu County at these labs based on historical data of 2019 (prior to COVID-19 pandemic). *Percentage of samples from Kisumu = Number of samples from Kisumu/Number of total samples* Data source: https://nascop.org
AMPATH	13% = 28 711/216 176	
Walter Reed CDC	5% = 10 270/191 302	
POC hub queueing parameters (existing)		
Mean service rate (test per day)	12	According to the GeneXpert platform specs, one single round of testing consumes around 1.5 h. We assumed that in each working day, approximately 3 rounds are conducted, accounting for sample preparation time, breaks, etc. In each single round, the POC VL test machine has 4 cartridges. Thus, about 12 test samples can be completed in one single machine per day.
Number of servers (Number of 4-cartridge machines assigned for each hub)		
Hub 1: Jaramogi Oginga Odinga Teaching & Referral Hospital	4	Data source: personal communication with implementing partner director for HIV programmes in Kisumu County
Hub 2: Kisumu County Hospital	2	
Hub 3: Chulaimbo County Hospital	1	
Hub 4: Muhoroni County Hospital	1	
Hub 5: Nyakach County Hospital	1	
Hub 6: Ahero County Hospital	1	
Hub 7: Kombewa County Referral Hospital	1	
Percentage of samples processed by POC machine that are for VL testing	100%	GeneXpert machines can additionally process TB samples, early infant diagnosis samples, COVID samples, STI samples, etc. We allowed model users to decide the dedications of the proportion of samples that are for VL testing to reflect this feature.
POC hub queueing parameters (added)		
Number of added hubs	1 to 7	Based on conversations with policymakers and national laboratory staff, this range was chosen to reflect potentially feasible numbers of new hubs
Mean service rate (test per day) [Average number of VL samples processed at one VL test machine]	12	[See details earlier in this table for POC mean service rate].
Number of servers [Number of machines assigned for each new hub]	2	

a Represents baseline parameter settings used in Results section.

### Optimization model formulation and analysis

In this section, we describe the formulation of our optimization model in terms of its decision variables, objective and constraints. The model aims to optimize the total TAT for the whole testing system during batching, transportation and testing through re-arranging the referral network and assigning new hubs. Details of the mathematical expressions are in Supplementary Appendix 4.

#### Decision variables and objective

We have two types of decision variables: opening indicators and referral indicators. The opening indicators are binary variables (i.e. 0 or 1) aiming to determine the opening status of facilities. If a facility is a central lab, existing hub or assigned as an added hub, its indicator equals 1, if not, it equals 0. The referral indicators connect clinics with testing demand and potential service sites. If the referral indicator is equal to 1, it means the corresponding clinic sends their testing samples to that service site.

The objective of the model is to minimize the system-level total TAT of the VL testing samples, which is the summation of time from all 146 facilities. The TAT included three parts: the batching delay at the sending facility, the transportation time between the sending facility and the selected testing site and the waiting time at the testing site. The batching delay reflects the time during which a facility collects and collates samples prior to sending the samples to a processing lab or hub. In consultation with Kenya team members, we modelled the batching delay with three modes: daily, twice weekly or once a week. After the facilities send their samples to the central lab or hub, the transportation time would be estimated. The expected waiting time at the testing location includes time waiting in the queue and the time at service (e.g. sample entering processing and machine processing) for every hub and central lab. The waiting time is estimated by the queueing model.

#### Optimization constraints

For the optimization model, we have the following constraints. The first constraint ensures that each testing demand is met, and the samples are assigned to only one open facility. The second constraint ensures that for each central lab or potential hub, if it is closed, then no clinics would send samples to them; if it is open, then the total number of accepted samples should not exceed its capacity. The third constraint ensures that the total number of open facilities is equal to a pre-specified number.

For Scenarios 2 and 3, the opening indicator for the 3 central labs and 7 existing hubs are assigned as 1, which guarantees that the existing testing systems are incorporated into our model.

#### Practical and fairness considerations

We included three additional considerations in the model (Table 1). The first consideration is that some clinics cannot host POC machines due to infrastructure limits. To determine the qualifications of the clinics, we referred to the Kenya Package for Health level of clinics. Under the guideline, facility levels 1–5 are community facilities, health dispensaries, health centres, county hospitals and county referral hospitals, respectively. We decided to choose only levels 3–5 as candidates for hubs as a proxy for sufficient infrastructure to support POC machines.

The second consideration is that GeneXpert^®^ machines are multi-disease diagnostic platforms that can additionally process specimens for tuberculosis, early infant HIV, COVID-19 and sexually transmitted infection diagnostics. We allowed model users to decide the proportion of samples that are for VL testing vs other diagnostics to reflect this feature.

The third consideration is that due to fairness concerns, we wanted to make sure that each sub-county had at least one hub. This involved a presumed trade-off between the efficiency and the fairness of the system. Thus, in Section 3.1, we tested both the efficiency-only base case model and the model with fairness considerations for Scenario 3.

#### Solving the optimization model

The hub selection problem is a binary-integer programming problem with a nonlinear objective. In this study, we only optimized the batching delay time and the transportation time in the objective function. The expected waiting time in the system is then added after obtaining the minimum batching delay and transportation time when evaluating the performance of the optimized system. This was based on the following two reasons. First, large-scale nonlinear integer programming remains hard to solve with current commercial solvers. Second, compared with the other two time components, the expected waiting time is only a small fraction of the total time. Thus, only considering optimizing the batching delay time and the transportation time would still provide a near-optimal solution, and we considered it as the most practical way to generate solutions for policymakers using widely available and open-source solvers. We used OpenSolver (http://opensolver.org), an Excel VBA add-in that could extend the function of the built-in solver in Excel, to solve the optimization problem. OpenSolver is free, easy to install and does not have any artificial limits on the size of the problem.

### Decision-support excel tool

**Figure 1.**
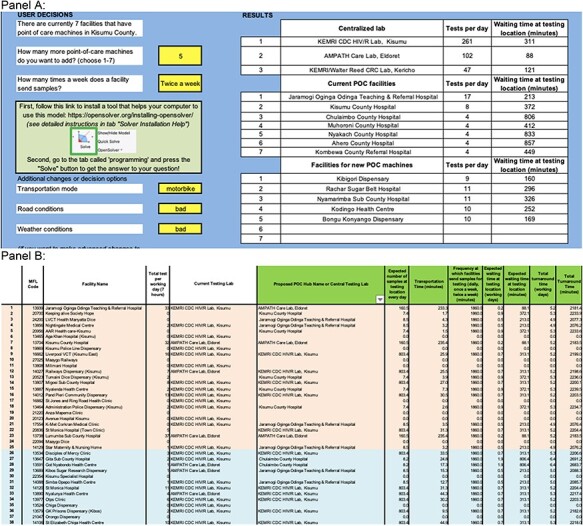
User interface of model with mock data. Panel A is the ‘Basic Inputs & Model Outputs’ tab. Panel B is the ‘Referral Network’ tab

We created an Excel tool that was intended to be user-friendly and usable on most computers without additional software requirements. We organized the model into eight tabs in Excel, classified into five categories based on their function. First, the ‘Basic Inputs & Model Outputs’ and ‘Advanced Inputs Changes’ tabs allow users to change the basic and advanced parameters and see the main results, including the hub names and expected waiting time at selected hubs and central labs (Figure 1, Panel A). The basic inputs tab was created to maximize usability for the broadest range of policymakers’ experience utilizing models. Second, in the ‘Referral Network’ tab, users could find detailed information for all 146 facilities, including the names, demand data, their current testing labs and the new testing locations to send their samples under the optimized hub-and-spoke model, and the simulated expected waiting time, transportation time and total TAT, etc. (Figure 1, Panel B). Third, in the ‘Programming’ tab, the users could see the way we lay out the optimization model and could also rerun the model using the OpenSolver package. Fourth, in the ‘M|M|s’ tab, we show the parameters for different queues; advanced users could change the service rate according to their local knowledge. In ‘M|M|s calculation’ tab, the users could see the detailed calculation and main output of each queueing system. Fifth, in the ‘distance_matrix’ and ‘transportation_time_matrix’ tabs, we provide the transportation data collected from Google Map, including the distance between each location and the estimated time for transportation. In order to maximize user-friendliness focused on simplicity, clarity and minimal opportunity to make an irrecoverable error, we created a ‘locked’ version of the model in which users cannot manipulate any data on the second through fifth categories of tabs.

## Results

### Formative input from stakeholders

Two virtual FGDs with 12 individuals were held in 2021 with various HIV treatment stakeholders, including representatives from county ministries of health and implementing partners, most with experience managing clinical teams that order or utilize VL testing results for patient management, some with experience coordinating laboratory logistics and some with experience in regulatory or budgetary decision-making (Table A2 in Supplementary Appendix 1: participant demographics).

In reflecting on the processes and factors that would influence their decisions of where to place POC machines for VL testing, participants described a wide range of considerations. They described engaging with decision-makers and civil society at multiple levels, including county assemblies, health committees and implementing partners in order to garner support for new technology. They described that decision making authority sat with programme officers, who then shared this with senior managers for discussion and approval. The range of factors that influenced their decisions included: staffing volume, capacity at the facility and training to run a test; geographic accessibility for patients and other facilities; disease prevalence, patient volume and infrastructure (e.g. electricity and back-up power). In prioritizing some facilities over others, they would consider high volume facilities (especially those with a large number of adolescents and young people failing to adhere to treatment), accessibility to other peripheral facilities and capacity to run tests with trained staff and finally those with the best laboratory and power infrastructure.

Participants endorsed all our a priori suggested model inputs and outputs and did not recommend dropping or modifying any factors. The inputs included: relative weight of decision factors, country guidelines, lab infrastructure (number of machines and capacity), sample transportation costs, centralized lab operating costs, POC operating costs and health impact. Of note, we operationalized costs and health impact in terms of time spent in the system and TAT rather than monetary costs. We incorporated the relative weight of decision factors by having two versions of the model, one with a fairness constraint and one without. The outputs included: sites recommended for POC machine placement, the number of tests performed by central labs vs POC sites and the machine types selected. Of note, we restricted to just one machine type for this modelling exercise. Participants requested the addition of a set of model outputs that described the clinical decision making impact of more timely results for both POC VL and drug resistance testing. This was beyond the capability of the proposed model and was not possible to incorporate.

The rest of this section is organized as follows: Section 3.2 provides a statistical summary of the performance of the system under different scenarios. In section 3.3, we visualize the facilities and referral networks on a map. Section 3.4 focuses on the sensitivity analysis for several important parameters, including distributional fairness of the system.

### Statistics summary of TAT

**Figure 2.**
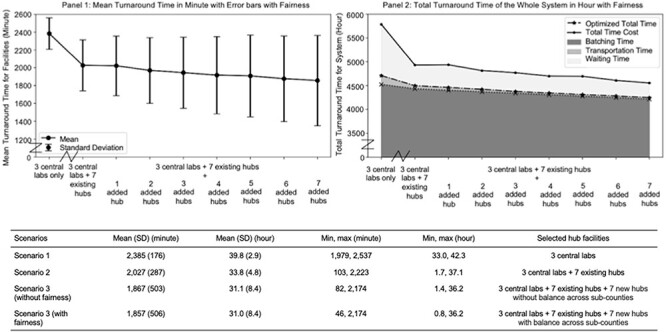
Panel 1: mean turnaround time in minutes for facilities with error bars with fairness considerations; table below provides specific numbers to accompany panel 1; Panel 2: total time in hours for the whole system with fairness considerations

We provide summary statistics of the TAT for all 146 healthcare facilities under different scenarios with the same parameter settings (Figure 2, Panel 2). All samples in the same facility are assumed to have the same expected TAT. The mean TAT is the average of the TAT per sample from all 146 facilities and is used to evaluate the facility-level performance under different conditions. As a baseline case, we assumed that the samples are sent twice a week by each facility under both average road and weather conditions. The transportation mode is assumed to be motorbike. For Scenario 3, we only show the result for 7 added hubs since it is the number with the lowest mean value of TAT. The full results can be found in Tables A3–A7 in the Supplementary Appendix 1.

Figure 2 illustrates the effect of different scenarios on the mean TAT for all 146 clinics. The left panel shows the facility-level effects: The steepest drop happens when the 7 existing hubs are added to the system. With an increasing number of added hubs, the mean TAT continues to decrease. The right panel shows the system-level effects. This plot breaks down the different components of time in the objective function. We note from the plot that the transportation time and batching time have a monotonically decreasing trend, while the waiting time generally decreases, but has some fluctuations when only a single hub is added to the system at each time. This is because we only optimized the batching and transportation time in the objective of the optimization model as described in the method section to reduce computational complexity.

### Referral network maps

**Figure 3.**
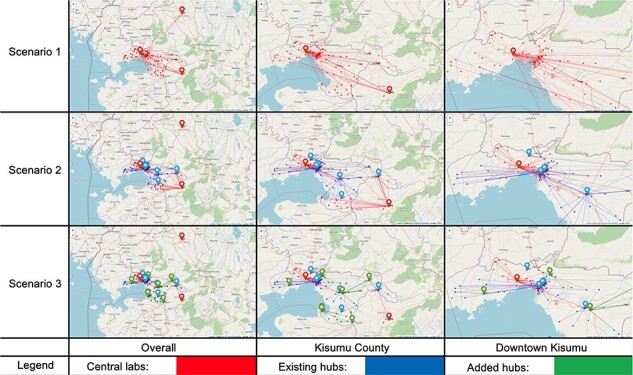
Referral network under different scenarios and zoom levels: Markers with colours of red, blue, and green correspond to central labs, existing hubs and added hubs, respectively. Dots representing 146 facilities with different colours correspond to the types of referral sites. The referral network is shown through links between facilities and selected testing sites

The referral network is visualized on the map in Figure 3. For each scenario, we show different zooming levels: one with the full map containing all facilities in Kisumu County and central labs outside the county; one with a zoomed-in map focusing on Kisumu County only; and one with a focus on a subregion in Kisumu (downtown area). Of note, the visualization results of the referral network for Scenario 3 assumes 7 added hubs and are under fairness constraints.

For Scenario 1, only three centralized labs are accepting samples. KEMRI CDC HIV/R Lab is located in Kisumu downtown, while the other two centralized labs are located outside the county. The AMPATH Care Lab has a long distance to the facilities in Kisumu. In addition, the figure also shows that under the current referral network, many facilities experience long transportation distances and cannot send their samples to the centralized lab closest to them. For Scenario 2, in general, clinics send samples to the centralized labs or hubs closest to them. The exceptions are due to limited capacity at their closest testing locations. In Scenario 3, we found that the furthest central lab (AMPATH Care Lab) is excluded from the referral network after more POC hubs are added to the system, due to its long distance to clinics in Kisumu County. This third central referral lab is not needed to meet VL testing demand for Kisumu County in a scenario with expanded POC availability, but could remain essential for other laboratory services.

### Sensitivity analysis

We conducted sensitivity analysis on several key parameters in our model. These parameters include: the operation capacity of centralized labs, number of machines in existing hubs or added hubs, transportation parameters and batching mode. Table 2 shows the detailed results of the sensitivity analysis, including the mean TAT of all clinics under all three scenarios with various model parameters. Of note, the Scenario 3 here also assumes 7 added hubs and with fairness constraints. We used the level of grey to reflect the change in terms of the mean TAT’s value under different parameter and scenario settings. We also computed the percentage change of mean TAT compared to the baseline result.

**Table 2.** Results for sensitivity analysis, mean turnaround time in minutes (sd) and percentage change from the baseline result

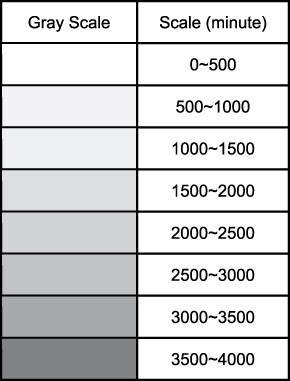

a Represents baseline parameter settings.

b Only Scenarios 2 and 3 have existing hubs.

c Only Scenario 3 has new additional hubs.

Note(s) Scenario 3 assumes the condition with 7 added hubs under fairness constraints.

The legend of the grey scale plot:
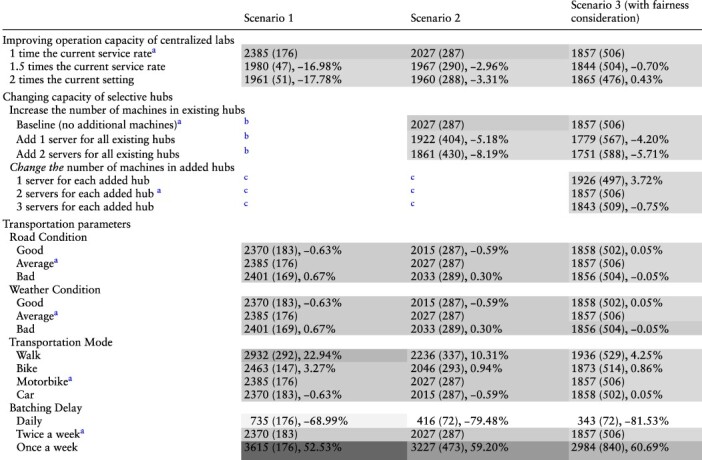

We found that the batching delay mode has the most drastic effect on the mean TAT. When changing the batching delay mode from twice a week to daily, the mean TAT drops by 68.99%-81.53% under different scenarios; when it changes from twice a week to once a week, the mean TAT increases by 52.53% - 60.69%. For the operational capacity in centralized labs, we found that it has a much larger effect under Scenario 1 than in subsequent scenarios that rely more on POC hubs. For example, when we increased the service rate in centralized labs to 1.5 times the current rate, the mean TAT drops by 16.98% under Scenario 1 but only drops by 2.96% and 0.70% for Scenarios 2 and 3, respectively. For the number of machines in existing hubs, results showed that Scenario 2 is more sensitive to this increase in the number of machines compared with Scenario 3; e.g., adding 2 servers for all existing hubs results in a reduction of 8.19% for Scenario 2 but 5.71% for Scenario 3. The road and weather conditions have similar and minor effects on the mean TAT under all scenarios (<1% change for each parameter under all scenarios). In contrast, the transportation mode affects TAT more substantially, particularly with walking sample delivery in Scenario 1.

## Discussion

In this study, we developed and tested a user-friendly Excel-based model intended for policymaker use in allocating a limited number of POC HIV VL testing machines as hubs within a geographic area and characterizing the optimal referral network. We found that increasing the number of POC hubs decreased the TAT, as expected. Specifically, the largest decrease in TAT was associated with moving from a scenario with only central laboratories to one with central laboratories and 7 existing POC hubs under an optimized referral network. However, the addition of 1–7 new POC hubs did further decrease the TAT, nearly monotonically, but with a lesser magnitude. The largest components of TAT were batching delays at the facility and waiting time at the lab or POC hub; transportation time was a substantially smaller component of overall time. Sensitivity analyses revealed that the largest reductions in TAT would be achieved if samples were batched more frequently (e.g. daily rather than twice weekly), if centralized laboratories expanded their operational capacity and if a faster mode of transportation was selected. Surprisingly, fairness constraints in new POC hubs allocations did not introduce markedly greater inefficiencies. Our findings suggest the following concrete activities for policy consideration: (1) utilizing existing POC machines that can conduct VL testing and (2) conducting operational improvements, such as batching and transporting samples, more frequently.

Our findings suggest that utilizing the existing POC machines—typically used for tuberculosis testing—to conduct HIV VL tests within a hub-and-spoke network of decentralized testing could markedly reduce TAT s. POC technologies are being scaled up globally, particularly in HIV diagnosis and treatment monitoring. Modelling has been used extensively to assess the impact of POC technologies on health and cost outcomes, including cost-effectiveness of POC vs traditional technologies for early infant diagnosis and VL monitoring (Simeon *et al.*, 2019; McCann *et al.*, 2020; Mukherjee *et al.*, 2020; Bulterys *et al.*, 2021; Salvatore *et al.*, 2021; Sharma *et al.*, 2021). Additionally, empirical studies focused on operational performance of POC technologies in real-world settings have considered the reductions in TAT and other operational performance (V. *et al.*, 2018; Drain *et al.*, 2020; Patel *et al.*, 2022). However, modelling studies that consider reducing TAT through optimal referral networks and POC placement are uncommon (Girdwood *et al.*, 2019; 2022). A recent abstract by Amick et al. considered the optimal placement of a fixed number of existing POC machines for HIV early infant diagnosis within a geographic network in Zimbabwe and assessed timeliness of infant HIV treatment initiation outcomes. They found that the functionality of machines was a major driver of overall performance and that optimizing the referral network, including the use of a fairness constraint, was beneficial for health outcomes (Yildirim *et al.*, 2022). These results are similar to our findings, including the finding that adding a fairness consideration only minimally reduced the efficiency of system performance.

Our model revealed that operational improvements—such as batching frequency, expanding the operational capacity of centralized laboratories and transport modality—had a larger impact than adding new POC machines. Technology-oriented solutions—such as POC machines—have demonstrated dramatic improvements in individual sites in trial and real-world contexts; for example, POC machines reduced TAT for early infant diagnosis in Mozambique from 127 to <1 days (V. *et al.*, 2018) and in six other African countries from 35 to 0 days (Boeke *et al.*, 2021b), POC machines reduced TAT for paediatric HIV VL monitoring in a Kenyan trial from 15 days to 1 day (Patel *et al.*, 2022) and in real-world contexts in 7 African countries from 65 to 6 days (Boeke *et al.*, 2021a). Such improvements of technological solutions drive many international donors to support the scale up of such interventions. However, modelling the impact of technology-oriented solutions within a real-world system can reveal unintuitive impacts and priorities. The impact of technology may be more modest or comparable to health system strengthening or operational changes. For example, a South African study identified that simply shifting the time of day when batched samples were collected by courier increased access to same day care by 6–13% in a modelling study (Girdwood *et al.*, 2022). Another modelling study in Zambia demonstrated that shifting to a transportation routing approach based on distance and volume rather than political borders could halve the cost of VL sample transportation (Nichols *et al.*, 2018). It was beyond the scope of this modelling exercise to compare the costs of operational vs added technology changes; future work could compare these approaches to maximize cost-efficiency.

While many modelling exercises aim at delivering a single optimized solution that is ideally adopted and enacted by policymakers, in this study, we aimed to create a tool that could be used and manipulated by policymakers to address their specific priorities and questions. Modelling exercises that are aimed at policy impact will have limited utility if not designed with and for policymakers and tested for usability. In this study, we included formative qualitative work with policymakers and are currently conducting usability interviews with this model and will present this in a future manuscript. We are not aware of any other studies that test the usability of resource allocation models with policymakers from resource-limited settings. Kenyan national laboratory policymakers have built and utilized models for resource allocation for early infant diagnosis POC machines *(Lusike, Wafula, Kingwara, Kenya POC Placement Model, 2018)*. The present modelling builds upon this country-led work, and initial feedback from decision makers indicates that this model will be utilized in resource allocation decisions going forward, with potentially an eye for moving it from a county- to country-level model.

This study benefited from several strengths. We conducted formative qualitative work with potential end users to determine the content and format of the model; we utilized routine Kenyan data for parameterization of the model when possible and worked in conjunction with Kenyan experts to parameterize and create assumptions within the model; we utilized an open source solver to avoid additional costs or ongoing internet connection. However, there were a number of limitations. The open source solver is an additional installation step and may make the model less feasible to use; we are assessing drivers of usability in our aforementioned ongoing usability interviews with policymakers. In initial consultations with policymakers about the face validity of the model, the model was generally found to be reasonable, but they noted that it would be politically unpalatable to have unequal distribution of POC machines between the sub-counties. In response, we included a fairness constraint in the revised model, which we noted had minimal impact on the overall TAT for samples. We utilized VL demand data from 2019 rather than a more recent year to avoid atypical VL demand in 2020–2022 due to COVID-19 and related VL testing reagent shortages. The model was developed prior to the new 2022 Kenyan HIV guideline changes; however, the changes to VL monitoring are minor and we do not expect that they substantially impact model functionality. As mentioned above, it was beyond the scope of this project to include cost or budget impact; this would likely be of high interest to policymakers to inform their decision-making and is a future area for expansion. However, a study that assessed the costs associated with expanding VL testing through POC testing in Zambia found cost savings of 6–35% associated with expanded POC machine use in hub and spoke networks (Girdwood *et al.*, 2019). Additionally, a Kenyan modelling study found that expanding use of POC machines for VL testing resulted in both cost savings and health benefits in terms of HIV and opportunistic infections averted (de Necker *et al.*, 2019). Finally, this model is limited to Kisumu County and would need expansion to be used nationally in Kenya. In order for the model to be expanded, it would require the acquisition of pertinent data, including testing demand, central laboratory capacity, clinic geospatial information and POC machine expansion plans. By integrating these data, the model could be applicable to additional Kenyan and beyond-Kenya settings. The current model assumes operational parameters specific to GeneXpert^®^ platforms, such as a run time of 90 min or modularity for the number of cartridges that can be processed at the same time. While the model could be modified to incorporate diverse assays (including SAMBA^®^ and other POC VL platforms), the development of tailored demand estimation for each test platform would be necessary.

## Conclusions

A stakeholder-informed resource allocation queueing model identified optimal POC VL machine hub allocations and referral networks. While adding POC machines would decrease TAT, larger reductions in TAT could be achieved by more frequent batching of samples and increasing capacity of existing central labs and POC VL hubs. Inclusion of fairness constraints in new POC hubs allocations did not meaningfully increase TAT.

## Supplementary Material

czad111_Supp

## Data Availability

The data underlying this article are available in the article and in its online supplementary material.

## Reflexivity statement

The authorship team is composed of researchers, students, policymakers and implementers based in the USA and Kenya. Five of the authors (4 women, 1 man) – including the 2 first authors and 2 senior authors – are based at a high income country university, identify as people of colour, are not Kenyan and have training in industrial engineering, epidemiology, medicine and anthropology. Seven of the authors (4 men, 3 women) are Kenyan and based in Kenya as national laboratory leaders, research team members (with qualitative interviewing and quantitative analysis expertise) and programme implementers. As several members of the team were not Kenyan, the team took steps to engage collaboratively during the process of generating the research question, grant development and administration, data collection and interpretation and manuscript writing. In particular, the authors responsible for model building crafted scenarios based on policy-relevant questions from Kenyan national laboratory leaders. Additionally, the team engaged in ad hoc meetings throughout the project to ensure that Kenyan team members’ perspectives and priorities were centred in the research process and that the model had face validity and relevance for Kenyan policymakers.
