# Supplementary material for: Using queueing models as a decision support tool in allocating point-of-care HIV viral load testing machines in Kisumu County, Kenya
Source: Health Policy Plan. 2023 Nov 9;39(1):44–55. doi: 10.1093/heapol/czad111 (PMC10775219; doi:10.1093/heapol/czad111)
Supplement: czad111_Supp [file czad111_supp.zip › 11.14.22 Appendix .docx]

**Appendix**

**A1. Supplementary tables**

Supplementary Table 1: Scenario setting

| Scenarios | Central labs | Existing POC hubs | Added POC hubs | Optimization Involved? |
| --- | --- | --- | --- | --- |
| Scenario 1 | 3 central labs* | Not included | No added hubs | No |
| Scenario 2 | 3 central labs* | 7 existing hubs** | No added hubs | Yes |
| Scenario 3 | 3 central labs* | 7 existing hubs** | 1~7 added hubs | Yes |

*3 central labs: KEMRI CDC HIV/R Lab,  Kisumu, AMPATH Care Lab, Eldoret, KEMRI/Walter Reed CRC Lab, Kericho

**7 existing hubs: Jaramogi Oginga Odinga Teaching & Referral Hospital, Kisumu County Hospital, Chulaimbo County Hospital, Muhoroni County Hospital, Nyakach County Hospital, Ahero County Hospital, Kombewa County Referral Hospital

Supplementary Table 2: Demographics of FGD participants

| **Characteristic** | **Median (IQR) or n (%)** |
| --- | --- |
| Age | 43 (37.5, 45) |
| Workstation | |
| County MOH | 11 (92%) |
| Implementing partner | 1 (8%) |
| Male | 8 (67%) |
| Highest level of education | |
| Bachelor’s degree | 6 (50%) |
| Master’s Degree | 6 (50%) |
| Years of education completed | 20 (19, 22) |
| Years working with HIV treatment monitoring | 10 (9,13) |
| Activities related to HIV treatment monitoring currently involved in: | |
| Managing clinical teams that order or utilize drug resistance results for patient management | 11 (92%) |
| Managing clinical teams that order or utilize viral load results for patient management | 9 (75%) |
| Coordinating logistical issues for HIV laboratory tests | 3 (25%) |
| Regulatory, validation, or verification of HIV-related machines or procedures | 3 (25%) |
| Determining budgets | 2 (17%) |
| Ordering and interpreting viral load for patients | 1 (8%) |
| Ordering and interpreting drug resistance tests for patients | 1 (8%) |
| Other coordination | 1 (8%) |

Supplementary Table 3: Mean turnaround time in minute with each additive hub for scenario 3 without fairness considerations

| Number of added hubs | Mean (SD) | Min, max | Selected hub facilities |
| --- | --- | --- | --- |
| 1 | 2,003 (333) | 103, 2,231 | 3 central labs + 7 existing hubs + List 1 |
| 2 | 1,981 (371) | 101, 2,229 | 3 central labs + 7 existing hubs + List 2 |
| 3 | 1,955 (410) | 56, 2,234 | 3 central labs + 7 existing hubs + List 3 |
| 4 | 1,917 (428) | 60, 2,174 | 3 central labs + 7 existing hubs + List 4 |
| 5 | 1,891 (468) | 60, 2,222 | 3 central labs + 7 existing hubs + List 5 |
| 6 | 1,881 (484) | 49, 2,201 | 3 central labs + 7 existing hubs + List 6 |
| 7 | 1,867 (503) | 82, 2,174 | 3 central labs + 7 existing hubs + List 7 |

List 1: Nyamarimba Sub County Hospital

List 2: Nyang'oma Sub County Hospital, St Clare Bolo Health Centre

List 3: Rachar Sugar Belt Hospital, Nyamarimba Sub County Hospital, Oren Health Centre

List 4: Tamu Health Centre, St Clare Bolo Health Centre, Ahero Medical Centre, Rodi Health Centre

List 5: Kibigori Health Centre, Rachar Sugar Belt Hospital, Sigoti Health Centre, Sango Rota Health Centre, Awasi Mission Health Center

List 6: Kibigori Health Centre, Nyang'oma Sub County Hospital, Nyabondo Mission Hospital, St Clare Bolo Health Centre, Nyakongo health Centre, Arito Langi Health Centre

List 7: St Elizabeth Chiga Health Centre, Mashambani Health Centre, Katito Sub County Hospital, Nyamarimba Sub County Hospital, Kinasia Health Centre, Nyangande Sub County Hospital, Nduru Kadero Health Centre

Supplementary Table 4: Mean turnaround time in minute with each additive hub for scenario 3 with fairness considerations

| Number of added hubs | Mean (SD) | Min, max | Selected hub facilities |
| --- | --- | --- | --- |
| 1 | 2,022 (335) | 102, 2,244 | 3 central labs + 7 existing hubs + List 8 |
| 2 | 1,970 (368) | 86, 2,222 | 3 central labs + 7 existing hubs + List 9 |
| 3 | 1,945 (398) | 102, 2,222 | 3 central labs + 7 existing hubs + List 10 |
| 4 | 1,917 (433) | 69, 2,239 | 3 central labs + 7 existing hubs + List 11 |
| 5 | 1,909 (459) | 93, 2,222 | 3 central labs + 7 existing hubs + List 12 |
| 6 | 1,877 (481) | 50, 2,219 | 3 central labs + 7 existing hubs + List 13 |
| 7 | 1,857 (506) | 46, 2,174 | 3 central labs + 7 existing hubs + List 14 |

List 8: Nyalunya Health Centre

List 9: Gita Sub County Hospital, Wanganga Health Centre

List 10: Nyalunya Health Centre, Kibigori Dispensary, Nyamarimba Sub County Hospital

List 11: St Elizabeth Chiga Health Centre, St Vincents De Paul Health Centre, St Clare Bolo Health Centre, Opapla Health Centre

List 12: Nyalunya Health Centre, Maseno University Health Centre, Tamu Health Centre, Nyabondo Mission Hospital, St Clare Bolo Health Centre

List 13: Simba Opepo Health Centre, Sigoti Health Centre, Sondu sub county Hospital, St Clare Bolo Health Centre, Ahero Medical Centre, Nyakongo health Centre

List 14: Simba Opepo Health Centre, Kibigori Health Centre, Sondu sub county Hospital, Sango Rota Health Centre, St Jane Nursing Home, Ahero Medical Centre, Miranga Sub County Hospital

Supplementary Table 5: Total turnaround time in minute for the whole system under different scenarios

| Scenarios | Total Transportation Time | Total Batching Time | Total Waiting Time |
| --- | --- | --- | --- |
| Scenario 1 | 8,878 | 271,560 | 64,594 |
| Scenario 2 | 3,993 | 265,980 | 25,829 |
| Scenario 3 | 1,909 | 252,960 | 20,765 |
| Scenario 3 (with fairness) | 2,014 | 252,960 | 18,339 |

Supplementary Table 6: Total turnaround time in minute for the whole system under Scenario 3 without fairness considerations

| Number of added hubs | Total Transportation Time | Total Batching Time | Total Waiting Time |
| --- | --- | --- | --- |
| 1 | 3,248 | 264,120 | 26,092 |
| 2 | 2,590 | 262,260 | 25,852 |
| 3 | 2,305 | 260,400 | 24,973 |
| 4 | 2,054 | 258,540 | 21,453 |
| 5 | 1,763 | 256,680 | 20,802 |
| 6 | 1,563 | 254,820 | 21,172 |
| 7 | 1,909 | 252,960 | 20,765 |

Supplementary Table 7: Total turnaround time in minute for the whole system under Scenario 3 with fairness considerations

| Number of added hubs | Total Transportation Time | Total Batching Time | Total Waiting Time |
| --- | --- | --- | --- |
| 1 | 3,629 | 264,120 | 28,409 |
| 2 | 3,130 | 262,260 | 23,448 |
| 3 | 2,288 | 260,400 | 23,506 |
| 4 | 2,234 | 258,540 | 21,148 |
| 5 | 2,062 | 256,680 | 22,998 |
| 6 | 2,198 | 254,820 | 19,525 |
| 7 | 2,014 | 252,960 | 18,339 |

**A2. Supplementary figures**


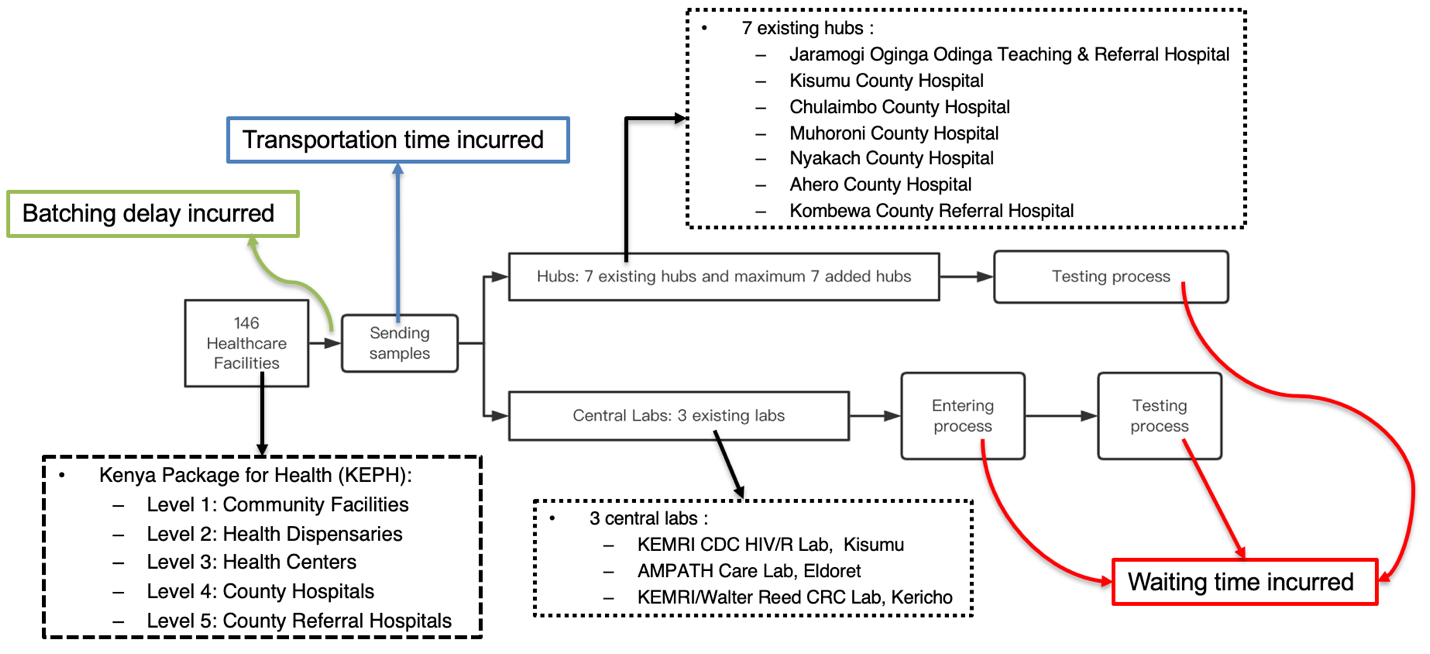


Supplementary Figure 1: Flowchart of the HIV VL POC testing system


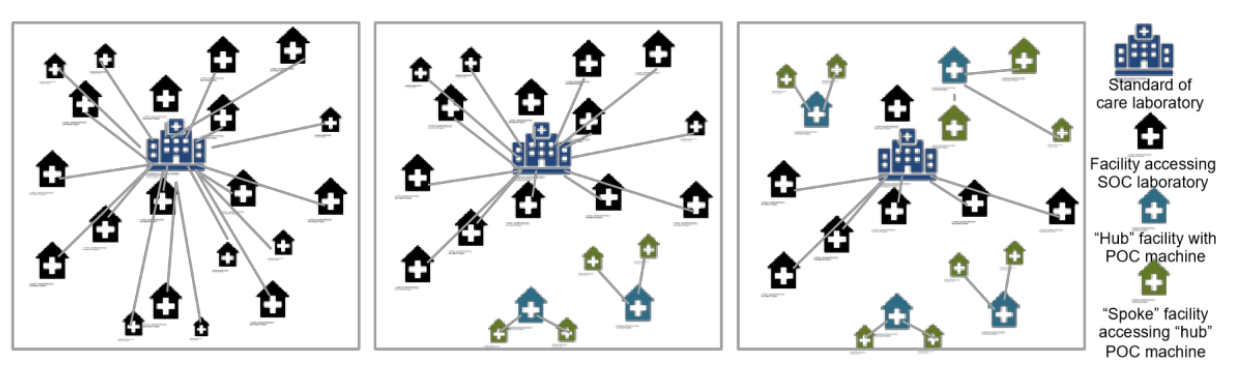


Supplementary Figure 2: Hub-and-spoke network structure with different scenarios: the left panel represents scenario 1, where only central labs are involved for testing; the middle panel represents scenario 2, where some hubs are added to the system; the right panel represents scenario 3, where more hubs are built in addition to the existing ones.


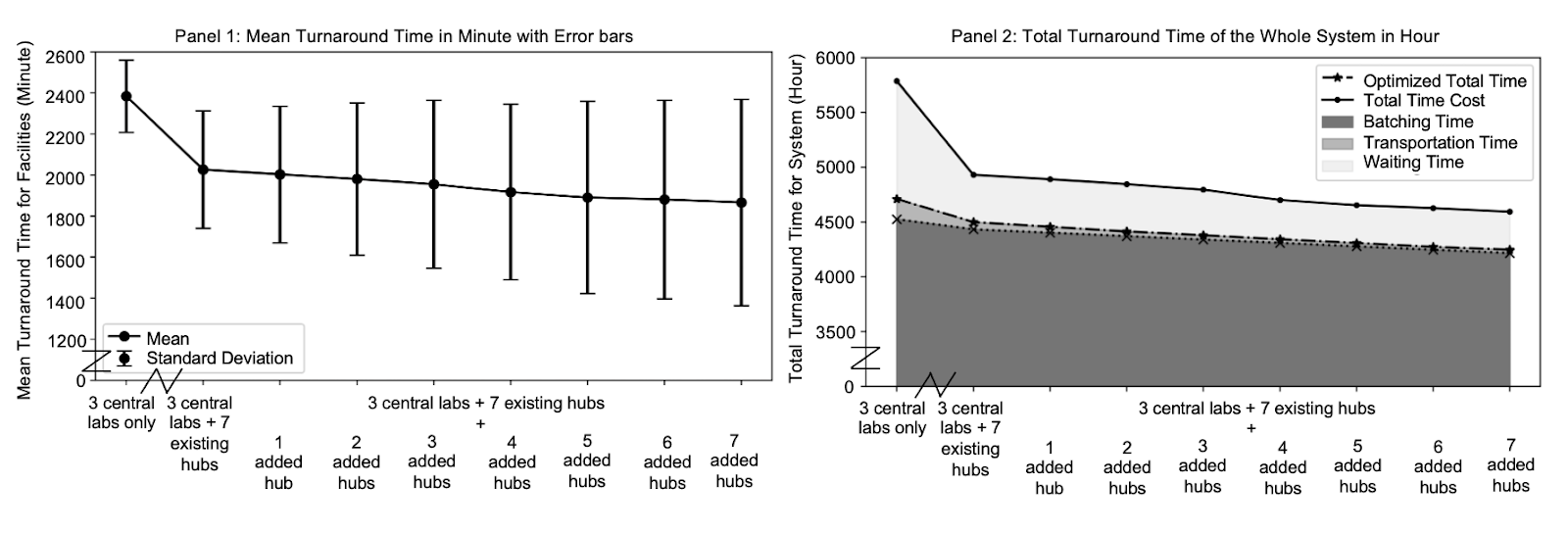


Supplementary Figure 3: Mean turnaround time for facilities with errorbars and total time cost for the whole system without fairness constraints

**A3. Mathematical Formulation of Queueing [1]**

M/M/s is one of the most widely studied queueing models [1]. M/M/s follows the ‘Kendall-Lee’ labeling convention, indicating that both the interarrival time distribution and service time distribution are Markovian (i.e., exponentially distributed), and the number of servers is s. Here we used a M/M/s queue to model the arrival and processing of VL testing samples at each selected hub in Kisumu County and at each central lab in Kenya. We assumed that the samples from each facility arrive at the hub with exponential interarrival times and the service time is also exponential. An M/M/s queueing model has an analytical solution, which means that once the mean arrival rate (number of samples arriving per unit of time), the mean service rate (number of samples tested per unit of time), and the number of servers (number of machines processing the samples in parallel) are specified we can calculate the expected time spent in the system. Details of the M/M/s queueing model can be found in Appendix 3.

**Notations**

$N(t)$: State of the system at time t: Number of customers in the queueing system (includes customers in service) at time t

$Q(t)$: Queue Length: Number of customers waiting for the service = State of the system – Number of customers being served

When the system is in steady state:

$P_{n}$: Probability that exactly n customers are in the queueing system

$L=\sum_{n=0}^{\infty} nP_{n}$: expected number of customers in the queueing system

$L_{q}=\sum_{n=s}^{\infty} (n-s)P_{n}$: expected queue length (excludes customers being served)

$\omega$: waiting time in the system (includes service time) for each individual customer

$W=E(\omega)$: expected time in the system

$\omega_{q}$: waiting time in the queue (excludes service time) for each individual customer

$W_{q}=E(\omega_{q})$: expected time in the queue

$\lambda$: mean arrival rate

$\mu$: mean service rate

$s$: number of servers

$\rho=\lambda/{(s\mu)}$: utilization ratio, and we require $\lambda<\mu$, that is $\rho<1$, otherwise, the queue would fail to reach steady state (e.g. the queue length will reach infinity in the long run). In addition, to avoid extremely large value for expected waiting time, we restrict the utilization ratio to be lower than 90%.

**Expected waiting time**

In addition, by Little’s Formula, we have:

Expected number in system = (Arrival rate) * (Expected time in system)

$$L=\lambda W$$

$$L_{q}=\lambda W_{q}$$

Expected time in system = (Expected time in queue) + (Expected time in service)

$$W=W_{q}+\frac{1}{\mu}$$

The idle probability $P_{0}$ could be expressed as follows:

$$P_{0}=\frac{1}{\sum_{n=0}^{s-1} \frac{{(\lambda/\mu)}^{n}}{n!}+\frac{\left( \lambda/\mu\right)^{s}}{s!}(\frac{1}{1-\rho})}$$

For $n\geq1$,

$$P_{n}=C_{n}P_{0}$$

where

$$C_{n}=\left\{ \begin{aligned} \frac{{(\lambda/\mu)}^{n}}{n!}, &n=1, 2,\ldots,s \\ \frac{{(\lambda/\mu)}^{n}}{s!s^{n-s}}, &n=s+1,s+2,\ldots\end{aligned} \right.$$

Then, for M/M/s queue, $L_{q}$ could be calculated as follows:

$$L_{q}=\frac{P_{0}{(\lambda/\mu)}^{s}\rho}{s!{(1-\rho)}^{2}}=\frac{P_{0}\lambda^{s+1}}{\left( s-1 \right)!\mu^{s-1}{(s\mu-\lambda)}^{2}}$$

And $W_{q}$, $L$, and $W$ could be calculated through Little’s Formula.

$$W=\frac{P_{0}\lambda^{s}}{\left( s-1 \right)!\mu^{s-1}{(s\mu-\lambda)}^{2}}+\frac{1}{\mu}$$

**A4. Mathematical Formulation of Optimization Model**

For modeling and optimizing the referral network, we let $I=146$ be the number of facilities collecting samples, and $J=I+3=149$ be the number of all potential hubs and central labs. To optimize the transportation cost through re-arranging the referral network and assigning new hubs, we formulate the following optimization problem. The mathematical formula of the optimization model is shown below.

**Notations**

$I = 146$: the number of clinics collecting samples

$J=149$: the total number of all 146 potential hubs and 3 central labs

$d_{i}$: the demand at $i$-th facility per working day (7 hours/day)

$B$: the batching delay time

$T_{ij}$: the transportation time from clinic $i$ to service (testing) site $j$

$k$: the total number of service sites that accept samples for testing

$U_{m}$: the index set of clinics in $m$^th^ sub-county

$W_{j}$: the expected time in service site $j$

**Decision Variables**

Since we want to optimize the referral network and select additional hubs, the decision variables are $x_{ij}$ and $y_{j}$.

$$x_{ij}=\left\{ \begin{aligned} 1, &\mathrm{if}i^{th} facility sends samples to j^{th}\mathrm{location} \\ 0, &\mathrm{otherwise} \end{aligned} \right.$$

$$y_{j}=\left\{ \begin{aligned} 1, &\mathrm{the}j^{th} location accept samples \\ 0, &\mathrm{otherwise} \end{aligned} \right.$$

**Objective**

The objective function is to minimize the total turnaround time of the system, including the total transportation time, total batching time, and total waiting time. Notice from the following objective, the total transportation time and total batching time are linear functions of the decision varibles, while the waiting time is a non-linear function of the decision varibles. The objective is shown by the formula below:

$$\min_{x_{ij},y_{j}} \sum_{i=1}^{I} \sum_{j=1}^{J} T_{ij}x_{ij}+\sum_{i=1}^{I} (1-x_{ii})B+\sum_{i=1}^{I} \sum_{j=1}^{J} x_{ij}W_{j}$$

For service site (hubs or central labs) $j$, the expected waiting time $W_{j}$ is:

$$W_{j}=\frac{P_{0j}{\lambda_{j}}^{s_{j}}}{\left( s_{j}-1 \right)!{\mu_{j}}^{s_{j}-1}{(s_{j}\mu_{j}-\lambda_{j})}^{2}}+\frac{1}{\mu_{j}}$$

Mean arrival rate:

$$\lambda_{j}= \sum_{i=1}^{I} d_{i}x_{ij}$$

Idle probability:

$$P_{0j}=\frac{1}{\sum_{n=0}^{s_{j}-1} \frac{{({\lambda_{j}}/{\mu_{j}})}^{n}}{n!}+\frac{\left( {\lambda_{j}}/{\mu_{j}} \right)^{s_{j}}}{s_{j}!}(\frac{1}{1-\rho_{j}})}$$

In the Excel tool, we only optimized the linear part of the objective due to computational complexities. This allows us to optimize the objective using Opensolver, an open-source Excel VBA add-in.

**Constraints**

In addition, we also have the following constraints:

| Mathematical formulations | Explanations |
| --- | --- |
| $\sum_{j=1}^{J} x_{ij}=1 \mathrm{for} i=1,\ldots, I$ | Each clinic only sends samples to one service site |
| $\sum_{j=1}^{J} x_{ij}d_{i}\leq s_{j}\mu_{j}y_{j} \mathrm{for} j=1,\ldots, J$ | Each clinic can only send samples to a selected site, and the total number of accepted samples in each selected site should not exceed its capacity |
| $\sum_{j=1}^{J} y_{j}=k$ | The number of opening service sites are pre-determined as $k$ |
| $y_{j}\leq Q_{j} \mathrm{for} j=1,\ldots, J$ | The choice of potential hubs is subject to their capacity and infrastructure qualifications (KEPH level)  $Q_{j}=\left\{ \begin{aligned} 1, &\mathrm{if}j^{th} facility has KEPH level of 3, 4, 5 \\ 0, &\mathrm{otherwise} \end{aligned} \right.$ |
| $\sum_{j\in U_{m}} y_{j}\geq1 \mathrm{for} m=1,\ldots, M$ | Due to fairness concerns, each sub-county in Kisumu should have at least one hub. |

In addition to the general constraints, we also need to preset the corresponding opening indicator $y_{j}$ as 1 for some testing sites. Specifically, for Scenario 2 and Scenario 3, the opening indicator for central labs and existing hubs are assigned as 1, which guarantees that the existing testing systems are incorporated into our model. Of note, in our Excel tool, we also set the maximum utilization as 0.9 to avoid overcrowding at the hubs or central labs, which may incur extremely long wait time.

**Reference:**

[1] Introduction to Operations Research, by Frederick Hillier, 10th edition, 2014.
